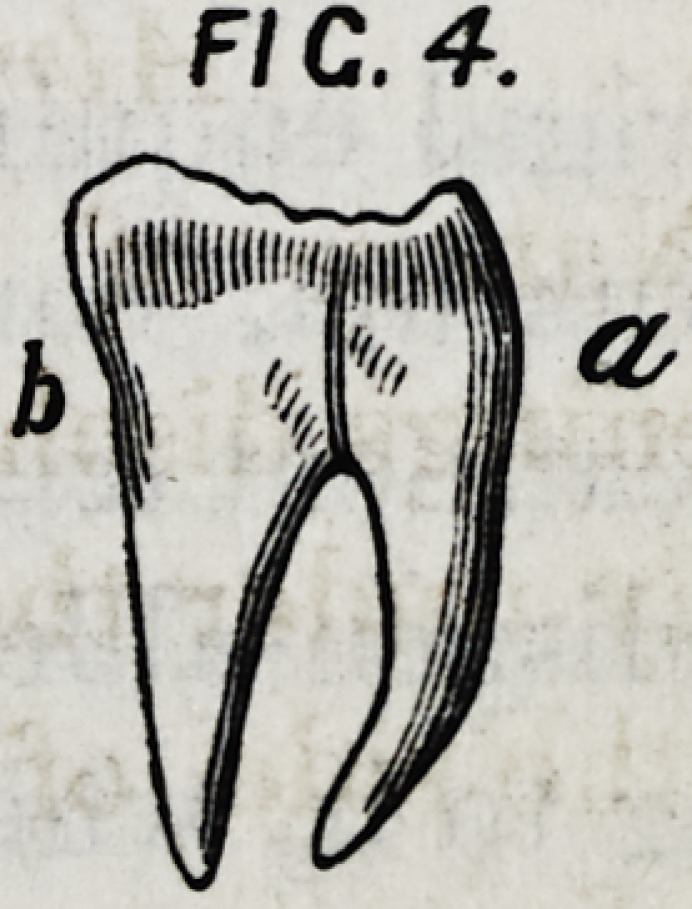# Notes from Dental Practice

**Published:** 1869-04

**Authors:** 


### ARTICLE III.

### *Notes from Dental Practice.*

*Filling Teeth.*—Cavity in the grinding surface of a superior molar. Nature of Case.—Cavity crucial in form, the decay extending from a central cavity along the crown fissures very nearly to the approximal surfaces on the one hand, and to the buccal and palatine surfaces on the other—very thin walls remained between the decayed fissures, which terminated in acute angles, and the surfaces named. See Fig. 1st.

Fig. 1.

Fig. 2.

Fig. 3.

Fig. 4.

Preparation of Cavity.—By means of a cone-shaped drill the central cavity, from which the fissure cavities in the first place proceeded, was enlarged, and the sharp, irregular projections of enamel forming angles at the points of union, together with the overhanging portions, partly removed, as is represented in Fig. 2.

By means of enamel chisels, the fissure cavities were then enlarged for some distance from the central cavity towards their extremities, the cavity at this stage of the operation having the form represented by Fig. 3.

When this was accomplished, a flat file, cut upon both sides, was applied by means of a file-carrier, first to the fissure extending very nearly to the buccal surface, and the thin wall intervening wholly cut away, opening out this fissure cavity on the buccal surface to a depth corresponding to that of the portion of the same cavity near to the central cavity, and giving to it a width of about one and a-half lines The opposite fissure, extending from the central cavity towards the palatine surface, was then enlarged in the same manner by means of the file, both as regards length, breadth and depth.

The file not being applicable to the fissures extending from the central cavity towards the approximal surfaces, on account of the presence of the adjoining teeth, enamel chisels were used to enlarge these fissure cavities to the same extent as were the fissure cavities extending towards the buccal and palatine surfaces, all the fissure cavities having, when prepared, perfectly parallel walls, and the form of the entire cavity, including central and fissure cavities, such as is represented in Fig. 4.

Filling the Cavity.—After carefully drying the cavity and protecting it from moisture by means of bibulous paper and napkins, the next step in the operation was the introduction of the gold—adhesive gold foil being used. Sheets and half sheets of the foil were formed into ropes from which pellets of different lengths were cut, and each pellet annealed previous to its introduction into the cavity. The first pellet, one of the largest size, was carried to the bottom of the fissure cavity extending towards the buccal surface, at its point of union with the central cavity. This pellet, owing to its size, when carried to the position named with the introducing plyers, and thoroughly condensed by means of mallet force, extended across the bottom of the fissure cavity and remained securely in place. Other pellets were then added to this and the bottom of the entire fissure covered, as far as the buccal surface of the tooth, the gold being built out a little beyond this surface for the purpose of properly finishing it. When this fissure was partly filled, the succeeding pellets were carried across the bottom of the central cavity, and from this cavity to the palatine fissure, which was partly filled in the same manner as the buccal fissure and central cavity. The gold was then introduced into the two approximal fissure cavities, anterior and posterior, and when these were partly filled, the operation of building towards the grinding surface was commenced and carried on until a sufficient quantity was introduced to completely fill the entire cavity, and restore the original form of the tooth.

### *Alveolar Abscess with Facial Fistulæ.*

Nature of Case.—Alveolar abscess with two fistulous openings discharging a very offensive matter, one immediately under the angle of the lower jaw, the other lower down on the side of the neck and about one inch to the front of the first.

Cause—Necrosed roots of an inferior second bicuspid and first molar. A probe introduced into the fistulous openings passed readily to the neighborhood of the apexes of the necrosed roots.

Treatment.—The necrosed roots were first extracted, and upon the apex of each was found adherent a portion of the sack of an alveolar abscess. After the copious discharge of blood mixed with pus, which followed the extraction of the roots, had somewhat subsided, the cavities were thoroughly syringed with tepid water, followed by a solution composed of

℞. Tinct. Iodine comp. gtt. xiv.

Acid Carbolic Cryst. (fusa). gtt. vj.

Glycerinæ . . . ʒ viij.

Aq. destillat. . . ℥ v. gtt.

This treatment was continued daily for about one week, a decided improvement taking place from the time the irritating causes were removed. The orifices of the fistulous openings presenting an unhealthy appearance, on the second day after the removal of the roots of the teeth, their edges were touched with a solution of nitrate of silver gr. ij. to ℥ j. of rose water, applied on a camel's hair brush.

### *Methods for Determining the Size of the Roots of Teeth previous to Extraction.*

By Mr. O. Salomon.

Before the educated dentist attempts the extraction of a tooth, he examines the form of the crown, which enables him to determine with certainty the direction of the roots. For young practitioners and students some indications will be of importance, therefore I give here those communicated by Dr. B. Whener:

FIG. 2.

FIG. 3.

FIG. 4.

I.—If the crown is large and short we may expect that the roots are long, while with a long and narrow crown the roots are small and slender.

II.—If the neck of a posterior tooth, *a* Fig. 1. is much thinner than its crown, the roots will diverge.

III.—If the neck of a posterior tooth, *b* Fig. 2. is as large as its crown, we may conclude that the roots run down parallel with the sides of the crown.

IV.—In case the neck of a posterior tooth, *c* Fig. 3. should be larger than the grinding surfaces, the roots will be found converging.

V.—When we observe one of the sides of the crown, *a* Fig. 4. inclining to the middle of the tooth, so we will find the corresponding root bent in the same direction, while the other roots (*c*) are found parallel with the perpendicular line of the tooth.

In the wisdom teeth the abnormal direction of roots is the most common.

## Figures and Tables

**Fig. 1. f1:**
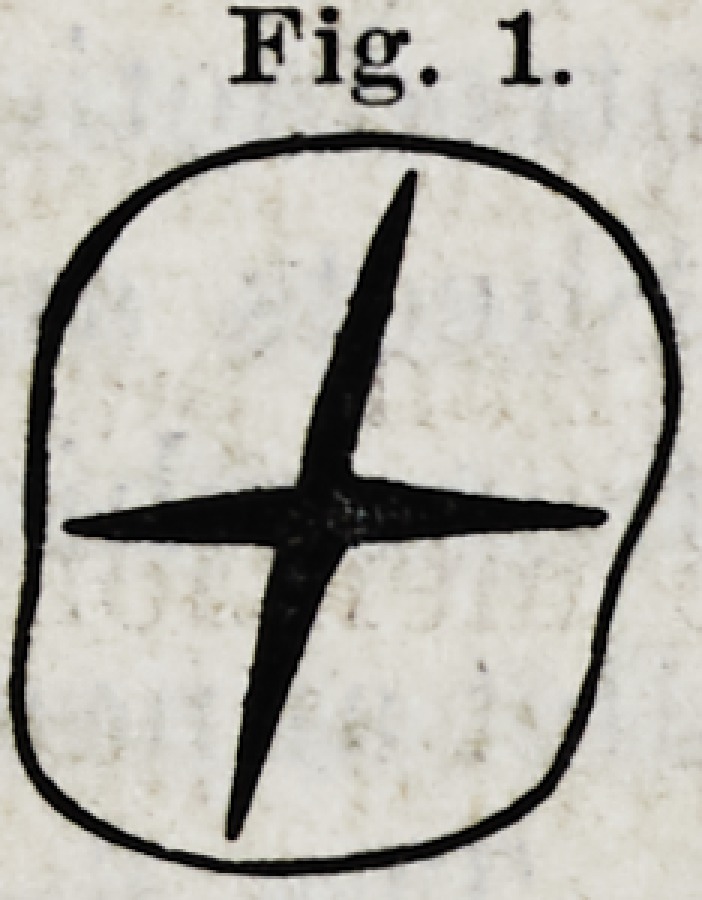


**Fig. 2. f2:**
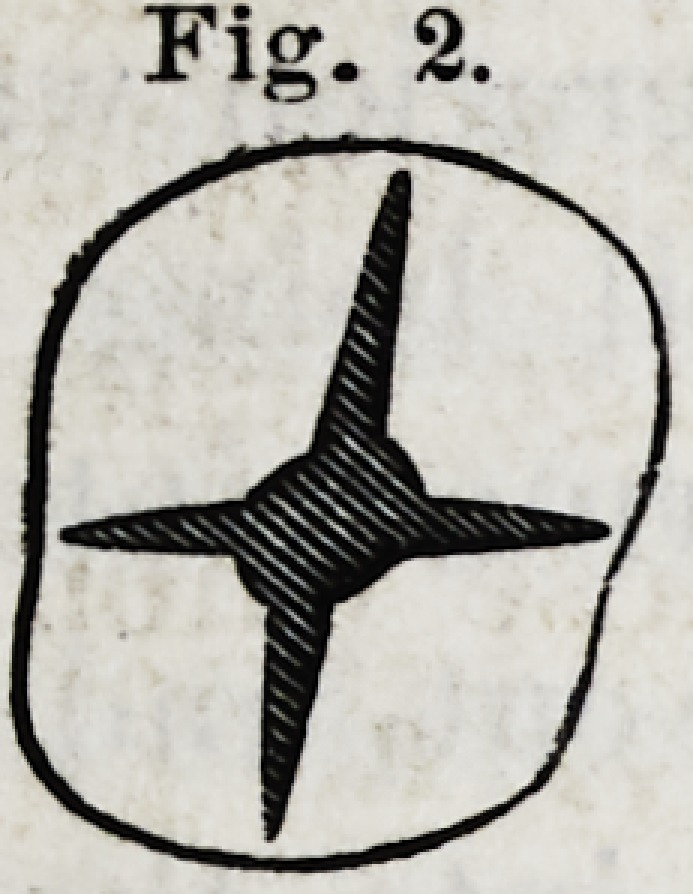


**Fig. 3. f3:**
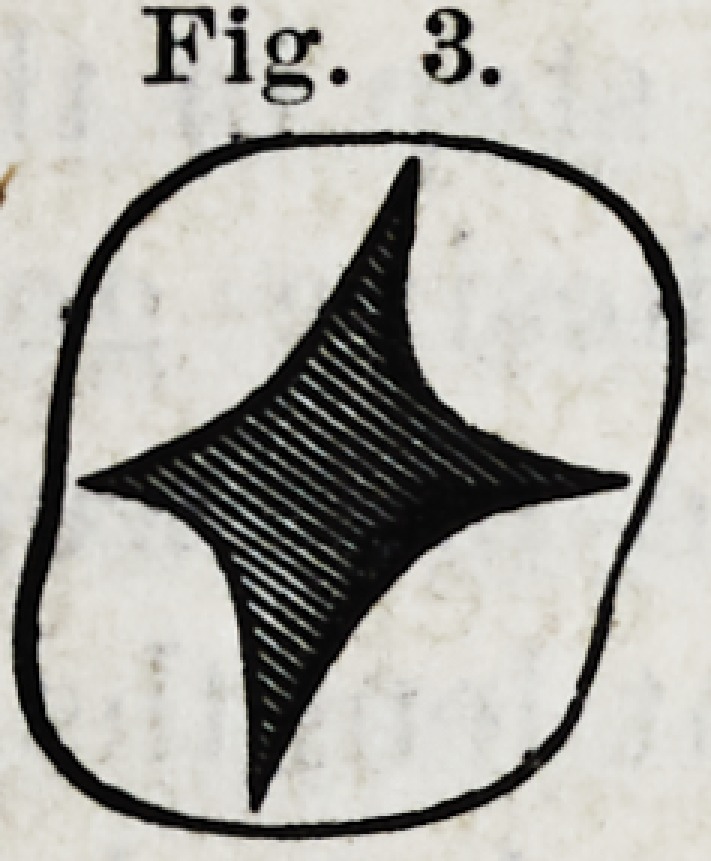


**Fig. 4. f4:**
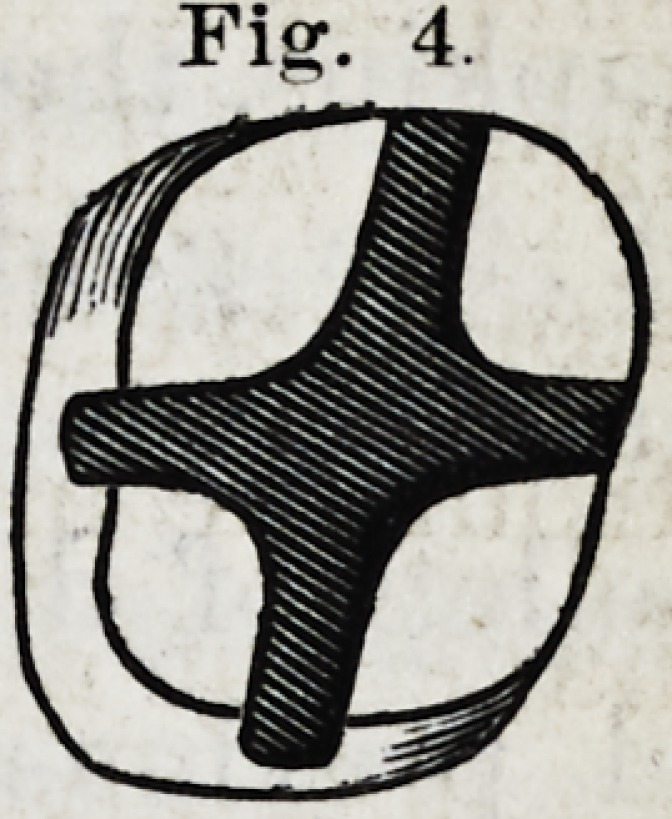


**Figure f5:**
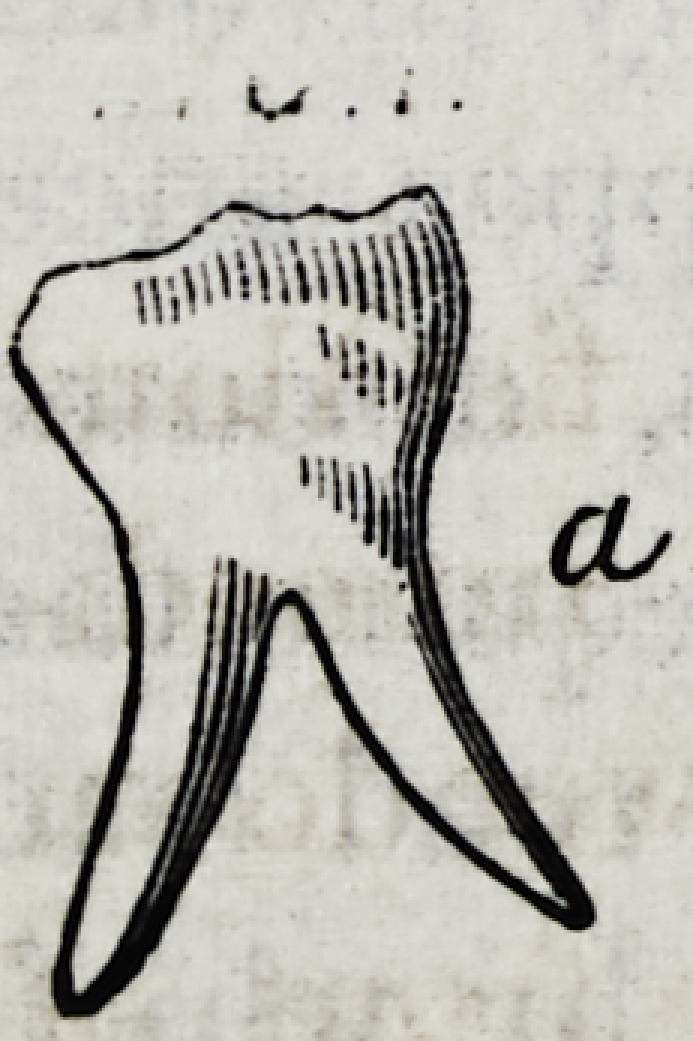


**FIG. 2. f6:**
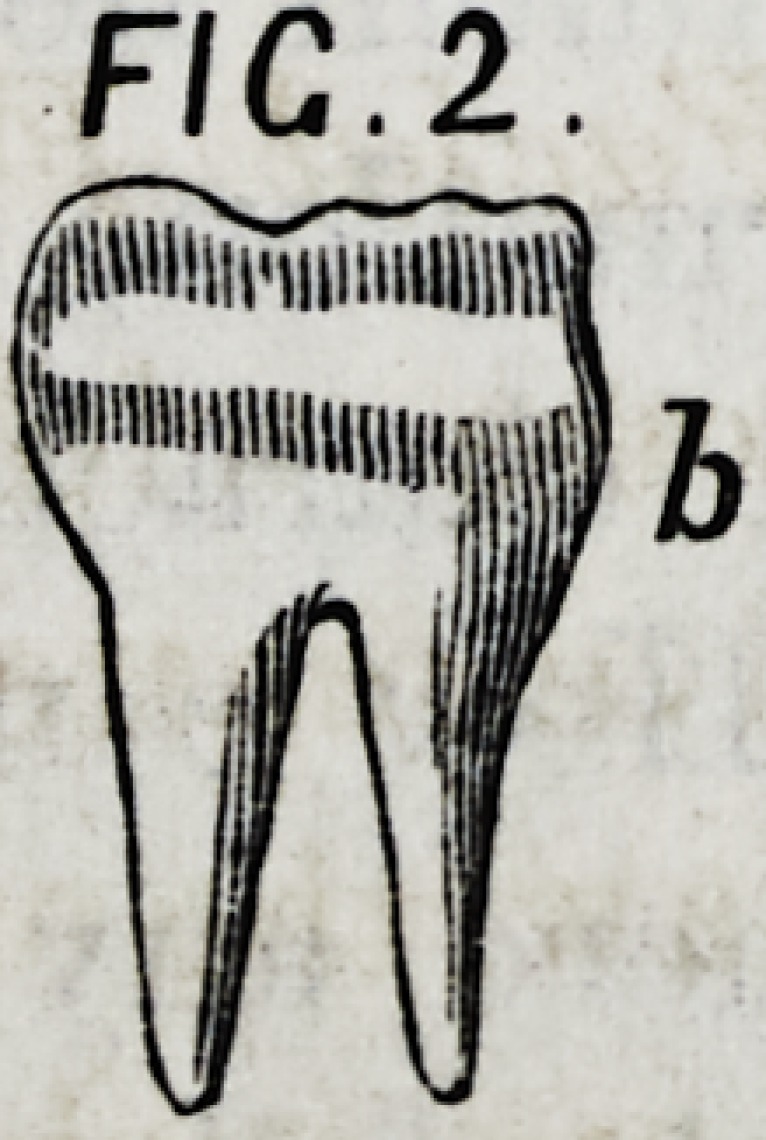


**FIG. 3. f7:**
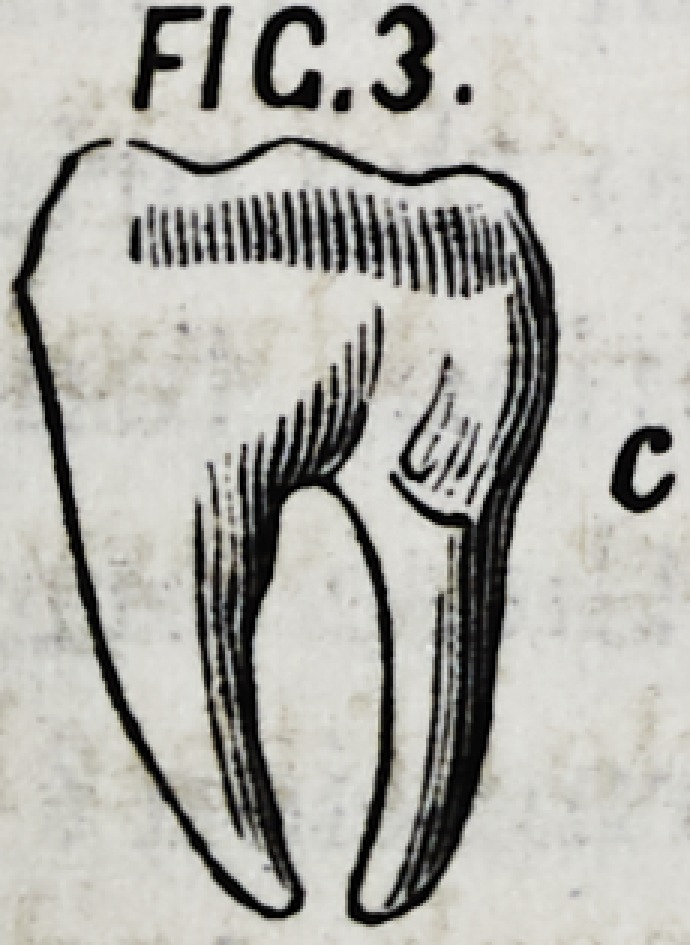


**FIG. 4. f8:**