# Erratum

**Published:** 1869-06

**Authors:** 


*Erratum.*—In the April No. of the *Journal*, under the head of "Notes from Dental Practice," in the recipe given for the treatment of Alveolar Abscess, instead of the abreviation gtt. at the end of the recipe, read *M.* or *Misce.* The error was due to the minim marks being changed to the drop marks after the proof was corrected.

As this is a valuable recipe we give it as it should read: ℞ Tinct. Iodine Comp. gtt. xiv. Acid Carbolic Cryst, (fusa) gtt.vj. Glycerinæ ʒ viij. Aq. Destillat, ℥ v. Misce.

It is well to state that after mixing, the dark color of the Comp. tinct. of iodine gradually disappears, and the solution at length becomes colorless, this change being entirely due to the carbolic acid. This solution possesses artificial and stimulant properties, and can be used in the form of injections, gargles, and lotions in diseases of the mucous membrane, ulcers, abscesses, &c.

